# Variation over Time in Child and Neighborhood Characteristics Associated with COVID-19

**DOI:** 10.1089/heq.2022.0213

**Published:** 2023-10-10

**Authors:** Sean Lang, Lori Silveira, Christiana Smith, Lisa Abuogi, Lisa Ross DeCamp

**Affiliations:** ^1^ Department of Pediatrics, University of Colorado Anschutz Medical Center, Aurora, Colorado, USA.; ^2^ Children's Hospital Immunodeficiency Program (CHiP), Children's Hospital Colorado, Aurora, Colorado, USA.; ^3^ Adult and Child Center for Outcomes Research and Delivery Science (ACCORDS), Aurora, Colorado, USA.

**Keywords:** SARS-CoV-2, health status disparities, census tract, pediatrics, immigrant health

## Abstract

**Introduction:**

To examine the associations between child and neighborhood characteristics and incidence of COVID-19 infection during the first 19 months of the pandemic.

**Study Design:**

We utilized individual electronic health record data and corresponding census tract characteristics for pediatric SARS-CoV-2 cases (age <18 years) from March 23, 2020 to September 30, 2021 with molecular tests resulted at a children's health system in Colorado. We compared associations between individual SARS-CoV-2 cases and census tract SARS-CoV-2 positivity rates over three time periods (TP1: March–September 2020; TP2: October 2020–March 2021; TP3: April–September 2021) using multinomial logistic regression for individual associations and negative binomial regression for census tract associations.

**Results:**

We included 7498 pediatric SARS-CoV-2 cases and data from 711 corresponding census tracts. Spanish preferred health care language was associated with SARS-CoV-2 positivity for TP1 (odds ratio [OR] 4.9, 95% confidence interval [CI] 3.7–6.5) and TP2 (OR 2.01, 95% CI 1.6–2.6) compared with TP3. Other non-English preferred health care language was associated with SARS-CoV-2 positivity in TP1 (OR 2.4, 95% CI 1.4–4.2). Increasing percentage internationally born in a census tract was associated with SARS-CoV-2 positivity for TP1 (multivariable incident rate ratio [IRR]=1.040, *p*<0.0001), TP2 (multivariable IRR=1.028, *p*<0.0001), and in all TP combined (multivariable IRR=1.024, *p*<0.0001).

**Discussion:**

Our study is notable for the identification of COVID-19 disparities among children in immigrant families and communities, particularly early in the pandemic. Addressing disparities for immigrant communities requires targeted investments in public health infrastructure.

## Introduction

The disproportionate impact of COVID-19 on racial/ethnic minority groups in the United States has been documented since the early months of the pandemic.^1^ African American, Latino, and American Indian/Alaska Native children and youth are at both higher risk of infection and more severe disease compared with White children.^2^ COVID-19 disparities reflect long-standing structural inequities for racial/ethnic minorities and low-income populations.^3–6^ Structural inequities increase exposures to negative social determinants of health (SDOH), which are well documented as contributors to health and health care disparities.^7–11^ Individual SDOH exposures often cannot be determined from epidemiological surveillance data.

However, contextual-level data, such as characteristics of the census tract or ZIP code, can provide information about potential SDOH exposures and inform our understanding of health disparities. Prior studies have demonstrated an association between contextual exposures to poverty and increased COVID-19 infection and mortality among adults in the United States.^12–16^ Adverse contextual exposures such as neighborhood poverty or low neighborhood opportunity are associated with worse child health outcomes.^17–20^ Information is lacking, however, about contextual exposures and COVID-19 infection among children.

As the COVID-19 pandemic has progressed, so has our understanding of preventive and mitigation measures. New vaccines and treatments have been developed and the response of local governments and the general public to national public health recommendations have become more varied. These changing dynamics during the COVID-19 pandemic underscore the need to examine disparities over time and across different geographies.^21^ Information on changing patterns of infection and severity could inform responses to future waves of infection and approaches to public health emergencies in the future.

Although COVID-19 has not enacted the same morbidity and mortality burden among children as adults, epidemiological information about children is still needed for preparedness due to their distinct needs and risks during public health emergencies.^22^ Therefore, the purpose of this study is to examine the individual and census tract characteristics associated with SARS-CoV-2 infection among children tested at the largest pediatric health system in the Mountain West region over three time periods (TP) during the initial 19 months of the COVID-19 pandemic.

## Methods

### Data sources

In this study, we utilized individual electronic health record (EHR) data and corresponding census tract level information for children <18 years old with a positive SARS-CoV-2 molecular test resulted at Children's Hospital Colorado (CHCO) locations between March 23, 2020 and September 30, 2021. CHCO includes a network of hospitals, emergency departments, and outpatient care centers serving ∼280,000 unique patients each year. Included cases had SARS-CoV-2 molecular tests obtained as inpatients, during outpatient clinic visits and at specialized drive-through testing centers located at health systems locations. Patients with more than one positive test were included based on the date of their first positive test. We used ArcGIS (Version 10.8.1.14362) to automatically assign the census tract using the home address listed in the EHR.

We included cases only for children located in geographic proximity to CHCO health system locations (Adams, Arapahoe, Denver, Douglas, El Paso and Jefferson counties). Cases with P.O. box addresses (*n*=48) were excluded from the sample as P.O. box locations may not accurately represent the characteristics of the child's place of residence. Geocoding addresses using the ArcGIS World Geocoding Service yielded an average matching score of 99.6. Census tract characteristics were determined using estimates from the 2015 to 2019 American Community Survey (ACS).^23^ Study activities were approved by the Colorado Multiple Institutional Review Board with a waiver of informed consent.

#### Individual measures

Child characteristics abstracted from the EHR included patient sex, age, race, ethnicity, preferred health care language and primary health insurance provider. Based on the race and ethnicity fields, we created the following mutually exclusive categories: American Indian/Alaska Native, Asian, African American/Black, Latino of any race, Native Hawaiian/Pacific Islander, White, Other/More than one race and Unknown. Owing to the volume of record creation for specialized testing centers the race/ethnicity field was often bypassed at registration by using an “Unknown” designation resulting in a relatively high proportion of unknown race/ethnicity. Other EHR characteristics were routinely completed and available for inclusion. We categorized preferred health care language as English, Spanish, or Other Language. Primary insurance was categorized as Public (Medicaid, Medicare, and Tricare), Private or Other (uninsured, charity care, and unknown).

#### Census tract measures

ACS estimates utilized in this study included: percentage non-White, percentage internationally born, percentage below the poverty level, and percentage crowding. These measures were selected based on emerging evidence of COVID-19 disparities for low-income people, immigrants, people of color, and in households with crowding.^1,24,25^ All census-tract covariates used in this data set are existing variables within the ACS summary data set. Persons in the following race/ethnicity categories are included in the census tract percentage non-White measure: Latino of any race, American Indian/Alaska Native, Asian, Black/African American, Native Hawaiian/Pacific Islander, and those who self-reported as more than one race or any “Other” race.^26^

Percentage internationally born corresponds to responses of “Outside the United States” to the question “Where was this person born?” The ACS uses questions on household size and income sources to determine the percentage of households below the poverty level based on threshold values set by the U.S. Census Bureau.^27^ The U.S. Census Bureau defines crowded households as those in which there are 1.01 or more individuals per room (not including bathrooms, patios, or crawlspaces).^28,29^

### Statistical analysis

In our analyses we compared associations between individual SARS-CoV-2 cases and census tract SARS-CoV-2 positivity rates over three TP: TP1 was March–September 2020; TP2 was October 2020–March 2021; and TP3 was April–September 2021. TP were constructed to have a similar number of months within each TP and to correspond to phases of the pandemic. TP1 corresponds to early pandemic, TP2 corresponds to the first winter surge, and TP3 corresponds to the initial Delta wave in the United States.

We used means and proportions to describe individual characteristics of cases by TP. The independent association of SARS-CoV-2 positivity with each individual-level sociodemographic characteristic over the three TP was compared using Proc GLM ANOVA's for continuous variables and generalized Fisher's exact tests for categorical variables. If a test for associations including all three TP was significant at the *p*<0.05 level, further evaluation was carried out using Student's *t*-tests or Fisher's exact tests to determine where differences existed between the three TP. In these cases, two-way comparisons were then completed comparing TP1 with TP2, TP1 with TP3, and TP2 with TP3. We then constructed a multinomial logistic regression model to examine associations between individual-level sociodemographic characteristics and SARS-CoV-2 positivity by TP. TP3 was chosen as the reference group as it had a sufficient number of cases and for ease of interpretation as it maintains chronological order.

To determine associations between census tract-level characteristics, we calculated the SARS-CoV-2 positivity rate for each tract based on individual cases and ACS tract population estimates for people <18 years. We used negative binomial regression modeling with a random effect for census tract to generate incident rate ratio (IRR) estimates of the association between SARS-CoV-2 positivity rates and included ACS measures. Tracts with zero or blank for the population counts for children <18 were not included in the analyses nor were tracts with missing values for the ACS variables that were included in the model.

The number of individual cases for each census tract was the outcome for the negative binomial model and an offset was included for the ACS tract population estimates for people <18 years to account for differences in tract size. For these analyses, we constructed four separate regression models for each ACS variable to complete univariable analyses and four multivariable models including all ACS variables. Each set of four models included one model for each TP and one that includes all TP. All analyses were performed in SAS 9.4 Copyright (c) 2002–2012 by SAS Institute, Inc. (Cary, NC).

## Results

We included 7498 pediatric SARS-CoV-2 cases in our analyses. Table 1 displays the sociodemographic characteristics of the cases overall and for each TP. Overall, the sample was 40.9% White and 29.4% Latino, 9.2% had preferred health care language other than English, and 43.6% had public health insurance. Results of multinomial regression analyses are presented in Table 2. Latino ethnicity was associated with greater odds of SARS-CoV-2 positivity for both TP1 (odds ratio [OR] 2.73, 95% confidence interval [CI] 2.26–3.30) and TP2 (OR 1.14, 95% CI 1.003–1.30) compared with TP3.

**Table 1.** Sociodemographic characteristics of children with positive SARS-CoV-2 tests

Category	Time period 1, March–September 2020 (***n***=894)	Time period 2, October 2020–March 2021 (***n***=4516)	Time period 3, April–September 2021 (***n***=2088)	All time periods, March 2020–September 2021 (***n***=7498)	***p*** ^a^
Sex, *n* (%)					0.31
Female	408 (45.6)	2188 (48.5)	1001 (47.9)	3597 (48.0)	
Male	486 (54.4)	2328 (51.5)	1087 (52.1)	3901 (52.0)	
Age, mean (SD)	8.9 (5.9)	8.8 (5.6)	8.1 (5.3)	8.6 (5.5)	<0.0001^b,c^
Race/ethnicity,^d^ *n* (%)					<0.0001^b,c,e^
American Indian/Alaska Native	3 (0.3)	4 (0.1)	9 (0.4)	16 (0.2)	
Asian	19 (2.1)	67 (1.5)	31 (1.5)	117 (1.6)	
African American/Black	39 (4.4)	171 (3.8)	182 (8.7)	392 (5.2)	
Latino (of any race)	419 (46.9)	1276 (28.3)	505 (24.2)	2200 (29.4)	
Native Hawaiian/Pacific Islander	4 (0.4)	7 (0.2)	3 (0.1)	14 (0.2)	
White	266 (29.8)	1934 (42.8)	875 (41.9)	3075 (40.9)	
Other/more than one race	33 (3.7)	182 (4.0)	102 (4.9)	317 (4.2)	
Unknown	111 (12.3)	875 (19.4)	381 (18.3)	1367 (18.3)	
Language, *n* (%)					<0.0001^b,c,e^
English	718 (80.7)	4103 (91.0)	1972 (94.7)	6793 (90.8)	
Spanish	148 (16.6)	347 (7.7)	83 (4.0)	578 (7.7)	
Other	24 (2.7)	58 (1.3)	27 (1.3)	109 (1.5)	
Insurance, *n* (%)					<0.0001^b,c,e^
Public	492 (55.0)	1827 (40.5)	948 (45.4)	3267 (43.6)	
Private	382 (42.4)	2630 (58.2)	1094 (52.4)	4106 (54.8)	
Other	20 (2.2)	59 (1.3)	46 (2.2)	125 (1.7)	

^a^ This *p*-value represents comparisons across all three time periods.

^b^ Comparison between time period 1 and time period 3 *p*<0.05.

^c^ Comparison between time period 2 and time period 3 *p*<0.05.

^d^ Race/ethnicity categories are mutually exclusive.

^e^ Comparison between time period 1 and time period 2 *p*<0.05.

SD, standard deviation.

**Table 2.** Relationship between SARS-CoV-2 positivity in each time period and individual sociodemographics using multinomial logistic regression

Category	Time period 1 vs. time period 3		Time period 2 vs. time period 3	
	OR (95% CI)	***p***	OR (95% CI)	***p***
Race/ethnicity (reference group: White)				
American Indian/Alaska Native	1.10 (0.30–4.08)	0.89	**0.20 (0.06–0.66)**	**0.008**
Asian	**2.02 (1.12–3.63)**	**0.02**	0.98 (0.63–1.51)	092
African American/Black	0.70 (0.49–1.02)	0.07	**0.43 (0.34–0.53)**	**<0.0001**
Latino	**2.73 (2.26–3.30)**	**<0.0001**	**1.14 (1.003–1.30)**	**0.04**
Native Hawaiian/Pacific Islander	4.39 (0.98–19.72)	0.05	1.06 (0.27–4.09)	0.94
Other	1.06 (0.70–1.61)	0.77	0.81 (0.63–1.04)	0.10
Unknown	0.96 (0.74–1.23)	0.74	1.04 (0.90–1.20)	0.60
Sex (reference group: female)				
Male	1.10 (0.94–1.28)	0.25	0.98 (0.88–1.09)	0.70
Age	**1.03 (1.01–1.04)**	**0.0003**	**1.03 (1.02–1.04)**	**<0.0001**
Language (reference group: English)				
Spanish	**4.90 (3.69–6.49)**	**<0.0001**	**2.01 (1.57–2.57)**	**<0.0001**
Other	**2.44 (1.40–4.26)**	**0.0017**	1.03 (0.66–1.63)	0.89
Insurance (reference group: Private)				
Public	**1.49 (1.27–1.74)**	**<0.0001**	**0.80 (0.72–0.89)**	**<0.0001**
Other	1.25 (0.73–2.13)	0.42	**0.53 (0.36–0.79)**	**0.0017**

Bold indicates *p*<0.05.

CI, confidence interval; OR, odds ratio.

Spanish preferred health care language was associated with greater odds of SARS-CoV-2 positivity for TP1 (OR 4.90, 95% CI 3.69–6.49) and TP2 (OR 2.01, 95% CI 1.57–2.57) compared with TP3. Other non-English preferred health care language was associated with greater odds of SARS-CoV-2 positivity in TP1 (OR 2.44, 95% CI 1.40–4.26) compared with TP3. Public health insurance was associated with greater odds of SAR-CoV-2 positivity in TP1 compared with TP3 (OR 1.49, 95% CI 1.27–1.74) and lower odds in TP2 compared with TP3 (OR 0.80, 95% CI 0.72–0.89).

Table 3 displays the characteristics of census tracts included in this study compared with Colorado census tracts overall. Included tracts had a slightly higher percentage of non-White, percentage internationally born residents and crowding and a somewhat lower percentage of residents below the poverty level compared with the state overall.

**Table 3.** Comparison of Colorado census tracts included in the study and all Colorado census tracts

Variables	Study census tracts (***n***=711)	All Colorado census tracts (***n***=1244)
	**Mean (95% CI)**	
% Non-White	34.4 (32.8–36.0)	31.1 (30.0–32.2)
% Internationally born	10.9 (10.86–10.94)	9.2 (8.78–9.62)
% Below poverty level	17.5 (16.8–18.1)	20.5 (19.9–21.1)
% Crowding	3.8 (3.29–4.31)	3.5 (3.12–3.88)

Table 4 displays the results of negative binomial modeling to determine the association between census tract characteristics and SARS-CoV-2 positivity. Univariable results demonstrated that increasing non-White percentage of the census tract was associated with a higher SARS-CoV-2 positivity rate for TP1 (IRR=1.010, *p*<0.0001) and in all TP combined (IRR=1.002, *p*=0.039). In multivariable analyses increasing tract percentage of non-White residents was associated with a decreased SARS-CoV-2 positivity rate in TP2 only (IRR=0.995, *p*<0.031).

**Table 4.** Relationship between census tract characteristics and SARS-CoV-2 positivity rate in children using negative binomial regression

Variables	Univariable incident rate ratio	95% CI	***p***	Multivariable incident rate ratio	95% CI	***p***
Time period 1						
Non-White	**1.010**	**1.006–1.013**	**<0.0001**	0.998	0.992–1.005	0.572
Internationally born	**1.031**	**1.023–1.038**	**<0.0001**	**1.040**	**1.025–1.055**	**<0.0001**
Below poverty level	**1.010**	**1.001–1.019**	**0.0039**	1.000	0.991–1.010	0.982
Crowding	1.009	1.000–1.019	0.054	0.989	0.977–1.001	0.073
Time period 2						
Non-White	1.000	0.99–1.002	0.72	**0.995**	**0.989–0.999**	**0.031**
Internationally born	1.006	0.999–1.013	0.060	**1.028**	**1.016–1.041**	**<0.0001**
Below poverty level	**0.990**	**0.984–0.997**	**0.0047**	**0.990**	**0.983–0.997**	**0.0095**
Crowding	**0.989**	**0.982–0.997**	**0.008**	**0.985**	**0.975–0.994**	**0.0015**
Time period 3						
Non-White	1.000	0.998–1.003	0.811	1.001	0.996–1.006	0.598
Internationally born	1.002	0.996–1.009	0.534	1.004	0.992–1.016	0.532
Below poverty level	**0.993**	**0.986–0.999**	**0.031**	**0.991**	**0.983–0.998**	**0.018**
Crowding	0.997	0.989–1.004	0.434	0.996	0.98–1.005	0.394
All time periods						
Non-White	**1.002**	**1.000–1.005**	**0.039**	0.997	0.99–1.002	0.326
Internationally born	**1.012**	**1.006–1.018**	**<0.0001**	**1.024**	**1.014–1.035**	**<0.0001**
Below poverty level	**0.992**	**0.986–0.998**	**0.0048**	**0.988**	**0.982–0.994**	**0.0002**
Crowding	0.997	0.990–1.003	0.386	**0.992**	**0.985–0.999**	**0.022**

Bold indicates *p*<0.05.

Both univariable and multivariable modeling demonstrated that an increasing percentage of internationally born in a census tract was associated with a higher SARS-CoV-2 positivity rate for TP1 (univariable IRR=1.031, *p*<0.0001; multivariable IRR=1.040, *p*<0.0001) and in all TP combined (univariable IRR=1.012, *p*<0.0001; multivariable IRR=1.024, *p*<0.0001).

In TP1 increasing percentage living below the poverty level was associated with a higher SARS-CoV-2 positivity rate in univariable modeling (IRR=1.010, *p*=0.0039). Conversely, an increasing percentage living below the poverty line was associated with a lower SARS-CoV-2 positivity rate for TP2, TP3, and all TP combined in both univariable and multivariable modeling.

## Discussion

This study provides novel information about individual sociodemographic and contextual associations with SARS-CoV-2 infection in children over time and offers important lessons for public health preparedness and ongoing management of current and future public health emergencies. We found that Latino ethnicity and Spanish preferred health care language were both associated with higher odds of SARS-CoV-2 infection in the first two study TP compared with TP3. In addition, a non-English non-Spanish preferred health care language was associated with higher odds of SARS-CoV-2 infection in TP1 compared with TP3, but not in TP2 compared with TP3.

These findings are consistent with other research related to the disparate impact of COVID-19 on Latinos and on populations with a non-English preferred language.^1,15,30,31^ Similarly, census tract multivariable analyses demonstrated a positive association between increasing percentage of internationally born residents in the tract and SARS-CoV-2 positivity earlier in the pandemic that decreased over time although remained an important factor across all TP combined. This, in combination with the individual results, suggests that immigrants and children in immigrant families were at particular risk of SARS-CoV-2 especially early in the COVID-19 pandemic.

Health disparities among racial/ethnic minorities are well documented, but there is comparatively less information on disparities among immigrant families.^24,32^ This may be due to challenges in data collection as administrative or epidemiological surveillance data may not provide enough information to identify immigrants or their family members.^32^ In this study, we used non-English preferred health care language as a proxy for a child in an immigrant family. There is a high level of correlation between non-English preferred health care language and limited English proficiency status.^33^ Most people in the United States with limited English proficiency are immigrants or children of immigrants.^34^

Higher incidence of SARS-CoV-2 among immigrants has been attributed to the higher proportion of immigrant workers in frontline essential workforce jobs that cannot be done from home as well as economic precarity decreasing the ability to take time off from work due to illness or exposure.^15,24^ Children in these families are thus more likely to be exposed to SARS-CoV-2 at home than children whose parents are able to work from home or take time off for illness or exposure. This may in part explain the associations in our study and in prior work documenting an association between SARS-CoV-2 positivity in children and non-English preferred language.^30^

Addressing and preventing disparities among immigrants presents challenges for public health preparedness. Availability of public health information in non-English languages has been found to be limited and when available often is the result of machine translation (e.g., Google translate), which is prone to errors.^35^ The time and costs associated with translation of materials, particularly when information was frequently changing during the early stages of the pandemic, may have proved an excessive burden for local health departments.

Although organizations such as the Centers for Disease Control and Prevention (CDC)^36^ began releasing material on COVID-19 safety and prevention in non-English language materials in the early months of the pandemic, the degree to which those resources were utilized by local public health agencies and/or accessed by individuals is unclear. In addition, CDC materials lack locally specific information on testing, vaccination, and other situationally tailored resource support. To increase the impact of public health media, repositories should include options for local customization, and public health agencies should work to optimize their access to and use of professional translation services.

Improving information access, although important, is unlikely to sufficiently address disparities. Immigrant families have reported racism and discrimination both generally and within health care systems before the pandemic and increases in racism and discrimination have been noted since the pandemic began.^37–40^ Addressing the impacts of racism and discrimination on the health and health care of immigrant families presents a complex challenge, but cannot be left unaddressed in efforts to promote health equity.

In this study, we also identified changes over time in COVID-19 disparities for Latino children. We found decreasing proportions of Latino children with SARS-CoV-2 among cases in TP2 and TP3. Decreasing racial/ethnic and income disparities over time among those affected by SARS-CoV-2 have been previously identified and have been attributed to expected pandemic epidemiology, whereby an increasing proportion of the population becomes infected over time, thus muting the impact of initial risk factors for infection.^41,42^

These findings could also indicate a changing approach to mitigation among different populations. Data suggest that racial/ethnic minorities were more likely than White populations to participate in mitigation activities such as masking and were more reluctant to have their children return to in-person school.^43–45^ Some studies, however, have noted that the trend of decreasing disparities reversed with the surge in cases associated with the Omicron variant, underscoring the need to maintain ongoing epidemiological surveillance to guide the public health response.^41^

Based on prior work we hypothesized that indicators of low socioeconomic status and greater household crowding in the census tract would be associated SARS-CoV-2 cases and tract positivity rates. Our findings demonstrate inconsistent associations between income and SARS-CoV-2 infection. In individual-level analyses, public health insurance was associated with higher odds of SARS-CoV-2 infection in TP1 compared with TP3 but lower odds in TP2 compared with TP3. In both univariable and multivariable census tract analyses starting in TP2 and overall, increasing tract poverty was associated with decreased SARS-CoV-2 positivity rates. Although worse health outcomes are commonly identified as income decreases, other studies have found similar inverse relationships between COVID-19 outcomes and contextual measures of income, and variability in these associations over time.^1,46^

Finally, contrary to our hypothesis, an increasing tract percentage household crowding was associated with decreased tract SARS-CoV-2 positivity rate for TP2 and overall. A positive association between household crowding and SARS-CoV-2 has been found in other studies, but associations have been found to vary over time during the pandemic and may be influenced by overall geographic area population density.^25,47^ Findings of a negative association in our study may reflect the geographic variation of included tracts and the multiple influences on household transmission, not all of which were evaluated in this study.

A notable limitation of our study is that changes over time may reflect changes in the tested population and/or access to testing rather than true changes in SARS-CoV-2 epidemiology. Reduced access to testing among low-income populations has been raised as a possible explanation for the seemingly paradoxical association with lower SARS-CoV-2 risk among low-income populations found in this and other studies.^48^ We did not have access to SARS-CoV-2 testing results and associated individual demographic data for testing performed outside the study health system, thus our findings may be specific to the populations who present to CHCO locations.

Early in the pandemic, however, the study health system was among a limited number of community testing sites and outpatient clinics in the region performing pediatric SARS-CoV-2 tests. Over time, the availability of pediatric testing at community sites and outpatient clinics increased. Low-income, racial/ethnic minority, and immigrant families may have sought testing at sites within their communities rather than at study testing sites. However, when we compared our case data with the race/ethnicity of pediatric COVID-19 cases in the state-wide database, the proportion of Latino children was similar (29.4% vs. 31%, respectively). Meanwhile, White and Black children were overrepresented in our data set compared with state SARS-CoV-2 case data (White children: 40.9% vs. 32%, Black children: 5.2% vs. 2.2%) [Race/ethnicity of testing data from the Colorado Department of Public Health and Environment; Gabrieloff E and Cronquist A, January 27, 2022], differences which may have biased our findings.

In addition, we did not use a composite measure such as the Child Opportunity Index^49^ as our contextual measure of neighborhood socioeconomic status. Although composite measures can provide additional important information about neighborhood features that contribute to children's health and well-being, the geographic areas included in this study have experienced rapid growth with rapidly changing neighborhood characteristics. Thus, we prioritized the use of more recent data than are available in the Childhood Opportunity Index as some components still rely on 2010 data. In addition, other research has shown stronger associations between COVID-19 and income than contextual socioeconomic composite measures.^1^

## Health Equity Implications

Our findings of COVID-19 disparities among Latino children reflect national trends among adults and indicate that continued efforts to support Latino communities in mitigation efforts including vaccination are needed to address COVID-19 disparities. We also found COVID-19 disparities among children in immigrant communities, indicating a critical need for investment in public health infrastructure specifically directed at these communities. Although our findings can inform public health preparedness, enhanced preparedness will not eliminate disparities during public health emergencies now or in the future. Long-standing inequities in health and opportunity in the United States will continue to drive disparate health outcomes if the structures that perpetuate the inequitable distribution of power and resources are not disrupted.

## Abbreviations Used

ACS: American Community Survey
ANOVA: analysis of variance
CDC: Centers for Disease Control and Prevention
CHCO: Children's Hospital Colorado
CI: confidence interval
EHR: electronic health record
GLM: general linear model
IRR: incident rate ratio
OR: odds ratio
SD: standard deviation
SDOH: social determinants of health
TP: time period
